# Therapeutic Potential of Combining PARP Inhibitor and Immunotherapy in Solid Tumors

**DOI:** 10.3389/fonc.2020.00570

**Published:** 2020-04-28

**Authors:** Praveen Vikas, Nicholas Borcherding, Adithya Chennamadhavuni, Rohan Garje

**Affiliations:** ^1^Department of Internal Medicine, College of Medicine, University of Iowa, Iowa, IA, United States; ^2^Holden Comprehensive Cancer Center, Iowa city, IA, United States; ^3^Department of Pathology, College of Medicine, University of Iowa, Iowa, IA, United States; ^4^Cancer Biology Graduate Program, College of Medicine, University of Iowa, Iowa, IA, United States; ^5^Medical Scientist Training Program, College of Medicine, University of Iowa, Iowa, IA, United States

**Keywords:** immunotherapy, PARP inhibitor, combination therapy, breast cancer, prostate cancer, gynecologic cancer, gastrointestinal cancers, solid tumors

## Abstract

Immunotherapy has revolutionized the treatment of both hematological malignancies and solid tumors. The use of immunotherapy has improved outcome for patients with cancer across multiple tumor types, including lung, melanoma, ovarian, genitourinary, and more recently breast cancer with durable responses seen even in patients with widespread metastatic disease. Despite the promising results, immunotherapy still helps only a subset of patients due to overall low response rates. Moreover, the response to immunotherapy is highly cancer specific and results have not been as promising in cancers that are considered less immunogenic. The strategies to improve immunotherapy responses have focused on biomarker selection, like PD-L1 status, and usage of combinatorial agents, such as chemotherapy, targeted therapy, and radiotherapy. Of particular interest, DNA-damaging agents have the potential to enhance the response to immunotherapy by promoting neoantigen release, increasing tumor mutational burden, and enhancing PD-L1 expression. Poly-ADP-ribose polymerase (PARP) inhibitors are one such class of drugs that has shown synergy with immunotherapy in preclinical and early clinical studies. PARP-based therapies work through the inhibition of single-strand DNA repair leading to DNA damage, increased tumor mutational burden, making the tumor a more attractive target for immunotherapy. Of the solid tumors reviewed, breast, ovarian, and prostate cancers have demonstrated efficacy in the combination of PARP inhibition and immunotherapy, predominately in *BRCA*-mutated tumors. However, initial investigations into wildtype *BRCA* and gastrointestinal tumors have shown moderate overall response or disease control rates, dependent on the tumor type. In contrast, although a number of clinical trials underway, there is a paucity of published results for the use of the combination in lung or urothelial cancers. Overall this article focuses on the promise of combinatorial PARP inhibition and immunotherapy to improve patient outcomes in solid tumors, summarizing both early results and looking toward ongoing trials.

## Introduction

A renaissance of cancer immunotherapy is currently underway for clinicians, researchers, and patients. Broadly speaking, cancer immunotherapy can be thought of as the selected manipulation of the balance between pro-tumor growth inflammation and anti-tumor immune responses (1). A number of different methodologies exist to shift the balance and promote anti-tumor immune response, most notably is immune checkpoint blockade, which act to remove the inhibition of anti-tumor lymphocytes (2). Checkpoint inhibitors (anti-PD-1/PD-L1 and CTLA-4 antibodies) are approved as single agent therapy and in combination with chemotherapy for a variety of cancers (3). Other mechanisms include targeted agents, personalized vaccines, T cell therapies, and dendritic-cell and non-specific agents, likes oncolytic viral therapy and modulators of the tumor microenvironment. Together, these immunotherapies may offer a chance at robust and durable responses for patients, however for the majority of non-immunogenic solid tumors, clear improvements in outcomes are still wanting (4).

**Figure 1:**
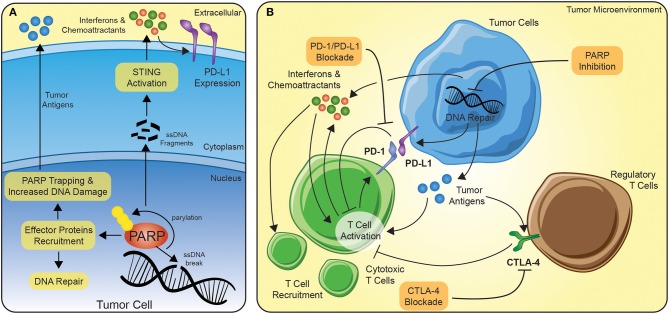
The potential synergy between PARP inhibition and immune checkpoint blockade. **(A)** In the context of PARP inhibition, tumor cells develop a more immunogenic repertoire of tumor antigens and release interferons/chemoattractants to serve as signals for immune cells. Interferons amplifies the immune activation of T cells and lead to further recruitment of T cells. **(B)** Immune checkpoint blockade can function on several levels, but principally the FDA-approved agents are directed at PD-1/PD-L1 or CTLA-4. PD-1 is transcriptionally upregulated on activating T cells and serve as a negative regulator of response. By disrupting the connection of PD-1 to PD-L1 expressed on tumor or stromal cells in the tumor microenvironment, the anti-tumor immune response can be sustained. Similarly, CTLA-4 is translocated to the cell surface of activating T cells or constitutively expressed on the cell surface of the suppressive regulatory T cells and dampens activation via the sequestration of co-stimulatory signals.

PARP inhibitors are more effective in tumors with existing defects in DNA damage repair (DDR), particularly, *BRCA1/2* mutations (5). In addition, PARP inhibitors are known to be more effective in tumors carrying somatic mutations in other DNA repair genes, including *ATM, ATR, BARD1, BRIP1, CHK1, CHK2, PALB2, RAD51*, and *FANC*, with defects in these genes might exhibit a phenotype similar to BRCAness (6). Functionally, as a DNA damage sensor, PARP enzymes are rapidly recruited to sites of single and double-stranded DNA damage (Figure 1A) (5). Binding to the DNA alters the catalytic domains of the PARP enzymes, leading to the production of ADP-ribose moieties (7, 8). The extension of the poly-ADP-ribose can serve multiple functions, including (1) recruiting effector proteins for DNA repair, (2) interfering with post-translationally modified chromatin proteins, like histones, and (3) can act as an energy sink for NAD+ molecules leading to cell death (5). PARP inhibition is not only thought to block proper DNA repair, but the inhibition can act to trap PARP at the replication fork, preventing transcription or translation (Figure 1A) (9).

In cancer cells with defective homologous recombination repair (HRR) and double-stranded DNA repair, PARP inhibition can lead to synthetic lethality, a combinatorial effect caused by deficiencies in multiple pathways (10). Most notably, this approach led to the recent Food and Drug Administration (FDA) approval of PARP inhibition for metastatic breast cancer (11) and as maintenance therapy for ovarian cancers (12), for individuals with germline mutations in *BRCA1* or *BRCA2*. Unlike the implicated role of PARP inhibition in facilitating cell death in cancer cells, new evidence is emerging in which PARP inhibition can enhance the response of immune checkpoint inhibitors (13, 14). PARP inhibition leads to the accumulation of DNA damage and by increasing the amount of DNA in the cytosol, PARP inhibition may trigger the interferon pathways (Figure 1A) (15, 16). Thus, not only can PARP inhibition lead to the accumulation of neoantigens for the anti-tumor immune response, but through the upregulation of interferons, the inhibition may act to prime the tumor microenvironment to facilitate a more profound anti-tumor immune response (Figure 1B). In addition, interferons have been shown to also increase expression of targets of immune blockade, like PD-L1, which suggests a further potential synergy between PARP inhibition and immune checkpoint blockade (17–19). Others have reported PARP-inhibitor-mediated upregulation of PD-L1 via interferon-independent mechanisms (20). This synergy may be the underlying mechanism of the marked preclinical responses seen using CTLA-4 and PARP inhibition in *BRCA1*-deficient tumors (21).

There exists a strong interplay among host immune system, DNA damage, and inflammation in the tumor microenvironment. Chronic PARP inhibition leads to sustained DNA damage resulting into epigenetic changes on tumor cells, hence making them more vulnerable to T cells and NK cells and finally resulting into increased intrinsic immunogenicity of tumor cells (22). Similarly, combinatorial trials utilizing epigenetic modulators, may increase therapeutic response by altering the expression of DNA repair genes (23). These strategies act to drive the balance of the tumor microenvironment toward anti-tumor immune response by recruiting immune and inflammatory cells while enhancing extrinsic immunogenicity. Combination of DNA damaging agents, like PARP inhibitors, and immune checkpoint blockers, may act as the key in bridging the potential of immunotherapies to a broader number of patients and tumors (Figure 1).

## Ovarian Cancer

Ovarian cancers represent a heterogenous group of solid tumors, that have had no major change in survival rates since the introduction of platinum agents (24). For example, relatively recent approval of VEGF inhibitor, like bevacizumab, with the standard carboplatin and paclitaxel chemotherapy was based on the demonstrated increased in progression-free survival improvement of 1.5 months (25). Part of the complications for the treatment of ovarian tumors vague constellation of symptoms leading to a late diagnosis (70% are stage III or greater) (26). Single allele hereditary mutations in DNA damage recognition or repair genes account for 24% of ovarian cancers (27). Of the one in five ovarian cancers associated with germline mutations, 65–85% are associated with germline *BRCA* mutations (28). Since the approval of PARP inhibitors in 2014 as a second line therapy for ovarian cancers (29), PARP inhibition has also been approved for maintenance therapy after response to platinum-based agents (30, 31). Phase III for the use of the combination as a maintenance therapy (NCT02477644) showed improvement in progression-free survival from 22.7 to 24.0 months compared to PARP inhibition alone (32). Additionally, the use of PARP inhibitors with bevacizumab in clinical trials (NCT02354131) have shown an impressive preliminary disease control rate of 92% in 12 patients with *BRCA2*-mutated ovarian cancers (33).

A number of clinical trials have examined or are currently investigating the synergistic effects of PARP inhibition and immune checkpoint blockade in ovarian cancer (Table 1). For example, the phase Ib basket study of the combination of anti-PD-1 antibody (BGB-A317) with PARP inhibitor (BGB-290) is currently underway for advanced solid tumors without *BRCA* mutation specifications. In the 34 patients which were treated, seven achieved a partial response (PR) and two women with ovarian cancer achieved a complete response (CR) (34). In the context of patients with known mutations in *BRCA1* or *BRCA2*, the phase II basket study (MEDIOLA) evaluated combination of durvalumab (anti-PD-L1) and olaparib in 32 patients with platinum-sensitive relapsed ovarian cancer (21). The combination of PARP and PD-L1 inhibition showed an overall response rate (ORR) of 63% (6 CR and 14 PR) and a 12-week disease control rate (DCR) of 81%. This combination was well-tolerated with grade 3–4 AEs of anemia (9%), increased pancreatic enzymes (6–9%), and neutropenia (3%). The larger TOPACIO trial showed more modest results in the evaluation of pembrolizumab (anti-PD-1) and niraparib in ovarian and triple-negative breast (TNBC) tumors with *BRCA* mutations vs. wild-type (wt) *BRCA1/2* (35). Across all 60 patients, there was an ORR of 25% and a DCR of 68%, with nearly a third of platinum-resistant ovarian cancers responding. Among the *BRCA*-mutated tumors, both ORR and DCR were elevated to 45 and 73%, respectively (35). Future trials, such as the ATHENA trial (Table 1), are evaluating the use of nivolumab (anti-PD-1) and rucaparib as maintenance following response to upfront platinum-based therapy in stage III/IV ovarian cancer (NCT03522246). The four arms will include: (1) maintenance nivolumab plus rucaparib, (2) nivolumab plus placebo, (3) rucaparib plus placebo, or (4) placebo alone. Likewise, the combination of PARP inhibition and checkpoint blockade with VEGF inhibition is being evaluated in several phase II trials (Table 1).

**Table 1:** Active combinatorial trials in gynecologic cancers.

**Trial ID**	**Cancer**	**PARP inhibitor**	**Immunotherapy**	**Phase**
NCT03101280	Ovarian + Endometrial	Rucaparib	Atezolizumab (PD-1)	I
NCT02571725	gBRCA ovarian	Olaparib	Tremelimumab (CTLA-4)	I/II
NCT02953457	•Ovarian •Fallopian tube •Peritoneal	Olaparib	Tremelimumab (CTLA-4)	I/II
NCT03572478	Recurrent Endometrial	Rucaparib	Nivolumab (PD-1)	I/II
NCT02660034	gBRCA Ovarian	BGB-290	BGB-A317 (PD-1)	I/II
NCT02657889	•Ovarian, •Fallopian tube •Peritoneal	Niraparib	Pembrolizumab (PD-1)	I/II
NCT02734004	Ovarian	Olaparib	Durvalumab (PD-L1) +/- bevacizumab	I/II
NCT02484404	•Ovarian, •Fallopian tube •Peritoneal	Olaparib	Atezolizumab (PD-L1) cediranib (VEGFR)	II
NCT03330405	Ovarian	Talazoparib	Avelumab (PD-1)	II
NCT03602859	Ovarian	Niraparib	TSR-042 (PD-1)	II
NCT03574779	Recurrent Ovarian	Niraparib	TSR-042 (PD-1)	II
NCT03955471	PLR Ovarian	Niraparib	TSR-042 (PD-1)	II
NCT03824704	•Ovarian, •Fallopian tube •Peritoneal •Serous Carcinoma	Rucaparib	Nivolumab (PD-1)	
NCT03574779	Ovarian	Niraparib	TSR-042 (PD-1) + bevacizumab	II
NCT02873962	•Ovarian, •Fallopian tube •Peritoneal	Rucaparib	Nivolumab (PD-1) + bevacizumab	II
NCT03694262	•Endometrial •Carcinosarcoma	Rucaparib	Atezolizumab (PD-1) + bevacizumab	II
NCT03651206	Carcinosarcoma	Niraparib	TSR-042 (PD-1)	II/III
NCT03737643	Ovarian (Maintenance)	Olaparib	Durvalumab (PD-L1) + bevacizumab	III
NCT03642132	Ovarian	Talazoparib	Avelumab (PD-1)	III
NCT03598270	Ovarian	Niraparib	Atezolizumab (PD-L1) + carboplatin	III

*gBRCA, germline BRCA mutation; PLR, platinum-resistant*.

## Breast Cancer

Breast cancer can be divided clinically by the expression of estrogen receptor (ER), progesterone receptor (PR), and the human epidermal growth factor receptor 2 (HER2). These receptors not only inform therapeutic options for a patient, such as selective estrogen modulators, but can act as biomarkers that predict disease course. The absence of these three receptors, a subtype of breast cancer referred to as TNBC, represents a poor prognosis for the respective 15–20% of breast cancers (36). Interestingly, 70–80% of TNBC are referred to as basal-like are defined by expression of basal cell markers and high levels of genomic instability, for example, p53 pathway alterations seen in 85–95% of tumors (36). This genomic instability in basal-like breast cancer (BLBC) is compounded by the characteristic loss in chromosome 5q, consisting of a number of DNA repair genes (36, 37). In addition, 15–20% of BLBC possess mutations in *BRCA1* or *BRCA2* (36, 38–40). Half of the 15–20% of BLBC tumors with *BRCA1* or *BRCA2* are a result of germline mutations (41, 42).

Breast cancer with germline mutations in *BRCA1* or *BRCA2* have been a focus of therapeutic targeting. Monotherapy PARP inhibitors have shown improved responses in germline *BRCA*-mutated breast cancer compared to conventional chemotherapy. The high ORR of 62.6% with talazoparib vs. control ORR of 27.2% in the phase III EMBARCA trial (NCT01945775) led to the FDA approval of the inhibitor for locally-advanced or metastatic HER2-negative breast cancer with germline mutations in *BRCA1/2* (43). Similarly, olaparib was FDA approved based on the results of the OlympiAD trial (NCT02000622) where the response rate was 59.9% in the olaparib group and 28.8% in the standard-therapy group (11). However, germline *BRCA* mutations only account for 8–10% of BLBC, with questionable efficacy for the majority of breast cancers without said germline mutations (36). Similarly, despite the increase immune infiltration of TNBC/BLBC compared to other breast cancers, the use of single-agent immune checkpoint blockade across all TNBC has found a wide range of objective responses from 5.4 to 33% (44). Thus, the combination of immunotherapy and PARP inhibition is currently being investigated (Table 2) for the promise of synergistic response in wild-type and mutant *BRCA* breast cancers (44).

**Table 2:** Active combinatorial trials in breast cancer.

**Trial ID**	**Cancer**	**PARP inhibitor**	**Immunotherapy**	**Phase**
NCT03101280	TNBC	Rucaparib	Atezolizumab (PD-1)	I
NCT03544125	Metastatic TNBC	Olaparib	Durvalumab (PD-1)	I
NCT02660034	TNBC	BGB-290	BGB-A317 (PD-1)	I/II
NCT02484404	TNBC	Olaparib	Atezolizumab (PD-L1) cediranib (VEGFR)	I/II
NCT02657889	TNBC	Niraparib	Pembrolizumab (PD-1)	I/II
NCT02734004	Breast Cancer	Olaparib	Durvalumab (PD-L1) +/- bevacizumab	I/II
NCT02849496	TNBC, stage III/IV	Olaparib	Atezolizumab (PD-L1)	II
NCT03167619	Advanced, platinum treated TNBC	Olaparib	Durvalumab (PD-1)	II
NCT03330405	TNBC	Talazoparib	Avelumab (PD-1)	II
NCT03740893	Advanced TNBC	Olaparib	Durvalumab (PD-1)	II
NCT03801369	Metastatic TNBC	Olaparib	Durvalumab (PD-1)	II

*TNBC, triple-negative breast cancer*.

As theorized, the combination of PARP inhibitor and immunotherapy has yielded promising initial response rates in both *BRCA1/2* mutated and *BRCA* wild-type (*BRCA*wt) patients. The phase I study evaluating durvalumab (anti-PD-L1 antibody) in combination with olaparib in 12 patients (10 with ovarian cancer and two with TNBC) with 11 of which were *BRCA*wt*, two* women achieved PR and eight women had stable disease, achieving an 83% DCR (45). The MEDIOLA phase I/II trial evaluated durvalumab and olaparib combination as a first- or second-line therapy. In 32 patients with germline *BRCA* mutations, the combinatorial therapy had an ORR of 53% and a 12-weeks DCR of 47% with minimal adverse events (46). These promising results are leading to the expansion of treatment arms in the MEDIOLA trial, especially with the expansion of *BRCA*wt TNBC patients (47, 48). Similar response was seen in the KEYNOTE-162/TOPACIO trial evaluating pembrolizumab and niraparib with an ORR of 29% patients, eight of the 12 patients with germline *BRCA* mutations. More intriguingly, ORR was 14.3–19.2% in *BRCA*wt TNBC, with results still forthcoming (49). This study also found, regardless of *BRCA* status, an ORR in 33% of PD-L1+ compared to 15% of PD-L1- tumors, suggesting PD-L1 may serve as a proxy marker for the combinatorial therapy, similar to the use in immune checkpoint blockade monotherapy (4, 49).

## Urothelial Cancer

Recent investigations of urothelial cancers have demonstrated pathogenic or likely pathogenic mutations in DNA repair in up to 15% of urothelial cancers (50, 51). Nearly a third of these mutations were seen in DNA mismatch repair (50). In a phase II clinical trial, the safety and antitumor activity of rucaparib was evaluated in patients with metastatic urothelial cancer irrespective HRR status. However, the study was closed prematurely as there was no adequate clinical response as determined by the independent data monitoring committee. Additional studies with rucaparib and olaparib are planned in a cohort selected for HRR mutations.

The potential synergy and efficacy of combination of PARP inhibitors and checkpoint inhibitors in urothelial cancers are currently being evaluated in multiple clinical trials. One such study is the BISCAY trial, a multi-arm, multi-drug, open-label, phase Ib study with one arm evaluating the safety and tolerability of olaparib as a monotherapy, or in association with durvalumab (anti-PD-L1), for the treatment of metastatic urothelial cancer in patients who have progressed on prior treatment and possess defects in DNA-repair genes (NCT02546661). A similar combination is evaluated in a phase II clinical trial as first-line for platinum-ineligible metastatic urothelial cancer (BAYOU, NCT03459846). Safety and efficacy of these approaches are yet to be reported (Table 3).

**Table 3:** PARPi and immunotherapy combination trials in urothelial cancer.

**Trial ID**	**Cancer**	**PARP inhibitor**	**Immunotherapy**	**Phase**
NCT02546661	Metastatic Urothelial Cancer	Olaparib	Durvalumab (PD-L1)	Ib
NCT03459846	First line, platinum-ineligible Metastatic Urothelial Cancer	Olaparib	Durvalumab (PD-L1)	II
NCT03869190	Locally advanced Urothelial Carcinoma	Niraparib	Atezolizumab (PD-L1)	Ib/II

## Prostate Cancer

The prevalence of mutations in the DNA repair genes involved in HRR in men with prostate cancer irrespective of age or family history has been estimated at 11–23% (52). In the Trial of PARP Inhibition in Prostate Cancer (TOPARP-A) study, olaparib monotherapy had a composite response (defined as either objective response as per RECIST 1.1 with modified PCWG2 recommendations, a decrease in PSA of ≥ 50%, or a circulating tumor cell count conversion from ≥5 cells per 7·5 mL blood at baseline to <5 cells per 7·5 mL blood) of 88% with a median survival of 13.8 months in a small cohort of heavily pretreated patients with metastatic castrate-resistant prostate cancer (mCRPC) with HRR gene mutation (53). In another study (TOPARP-B), 98 patients with mCRPC with DNA repair gene mutations and prior treatment with chemotherapy and novel antiandrogen therapy were randomized to receive treatment with either olaparib 400 mg BID (Ola400) or olaparib 300 mg BID (Ola 300). The composite response was seen in 54·3 and 39.1% in the Ola400 and Ola300 cohorts, respectively. The radiologic responses were seen predominantly in patients with *BRCA 1/2* when compared to *ATM* and *CDK12* gene mutations (54). The preliminary results of the phase III, randomized PROfound study evaluating the efficacy of olaparib in comparison to physician choice novel antiandrogen (pcNHA) in mCRPC with HRR mutations, were presented at the ESMO conference in 2019. In this study, an objective response rate (based on RECIST v1.1 + PCWG3) of 33% was seen in patients with *BRCA1, BRCA2*, or *ATM* mutations and treated with olaparib in comparison to pcNHA the ORR was 2.3%. When the response was assessed with the inclusion of other HRR gene mutations, the ORR with olaparib was 21.7%, suggesting differential response with *BRCA* vs. *non-BRCA* mutations. The radiographic PFS for those treated with olaparib was 5.82 months, and with pcNHA, it was 3.52 months. At the data cut off, the overall survival was 17.51 and 14.26 months, respectively. The most common adverse events with olaparib monotherapy were anemia, nausea, decreased appetite, and fatigue (55).

The efficacy of niraparib in patients with mCRPC after prior novel hormonal agent and chemotherapy was evaluated in the GALAHAD clinical trial (56). Patients were enrolled based on the presence of HRR gene mutations. In the preliminary results report at the ESMO conference in 2019, 46 patients had *BRCA 1/2*, and 35 had *non-BRCA* mutations. The objective response rates were high with *BRCA 1/2* when compared to *non-BRCA* mutations, 41 vs. 9%, respectively. The median PFS in the *BRCA 1/2* and *non-BRCA* cohorts were 8.2 and 5.3 months, respectively. The most common grade 3–4 side effects were myelosuppression, asthenia, and back pain.

The safety and antitumor activity of immunotherapy with olaparib was evaluated in early phase studies. In a phase II, open-label study of 17 patients with mCRPC regardless of HRR mutation status, the combination of durvalumab and olaparib was evaluated (57). Of the 17 patients, 9 had a prostate-specific antigen (PSA) decline of ≥50%, and four had a radiographic response by RECIST v.1.1 criteria. Genomic analysis of the responding patients showed 4 with germline and 2 with bi-allelic somatic alterations in *BRCA2* (58). Additionally, one responder had a monoallelic loss of *PMS2*, a mismatch repair gene, and with a separate responder having a monoallelic loss of *BRCA2*. The remaining two responding patients had no predisposing gene alterations detected. The most common grade 3 or 4 adverse events were myelosuppression, infection, and nausea (57, 58). In another study, Yu et al. evaluated the efficacy of pembrolizumab along with olaparib in a cohort of 41 patients with mCRPC and HRR wild-type (59). This combination showed a partial response of 7% and disease control rate (DCR) of 29% as per the RECIST v1.1 criteria. The median OS was 14 months with the most common adverse events being anemia, fatigue, and nausea.

PARP inhibitor monotherapy in prostate cancer with HRR gene mutations, especially *BRCA1/2*, has shown significant antitumor activity. The hypothesis for combining immunotherapy with PARP inhibitors is plausible; however, the data on safety and antitumor activity are still in the early stages. Numerous combination trials to address the safety and efficacy are ongoing (Table 4).

**Table 4:** Active combinatorial trials in prostate cancer.

**Trial ID**	**Cancer**	**PARP inhibitor**	**Immunotherapy**	**Phase**
NCT02484404	Metastatic CRPC	Olaparib	Atezolizumab (PD-L1) cediranib (VEGFR)	I/II
NCT02660034	Metastatic CRPC	BGB-290	BGB-A317 (PD-1)	I/II
NCT02861573	Metastatic CRPC	Olaparib	Pembrolizumab (PD-1)	I/II
NCT03572478	Metastatic CRPC	Rucaparib	Nivolumab (PD-1)	I/II
NCT03330405	CRPC	Talazoparib	Avelumab (PD-1)	II
NCT03338790	CRPC	Rucaparib	Nivolumab (PD-1)	II
NCT03834519	Metastatic CRPC	Olaparib	Pembrolizumab (PD-1)	III

*CRPC, Castration-resistant prostate cancer*.

## Lung Cancer

Lung cancers can be subdivided into two major categories by histological examination, small-cell (SCLC) and non-small-cell lung cancers (NSCLC). In the latter, immune checkpoint monotherapy has shown responses ranging from 12 to 45%, dependent on patient selection criteria (4). Several studies are currently aimed at the combination of immune checkpoint blockade and PARP inhibition in NSCLC (Table 5), however, most efforts for improving therapeutic response in NSCLC is the combination of checkpoint blockade with targeted therapies (60) or traditional chemotherapy regimens (61–63). Or similarly, the testing the synergy of PARP inhibition with targeted therapies (64) or chemotherapies (65).

**Table 5:** Active combinatorial trials in lung cancer.

**Trial ID**	**Cancer**	**PARP inhibitor**	**Immunotherapy**	**Phase**
NCT02484404	SCLC	Olaparib	Atezolizumab (PD-L1) cediranib (VEGFR)	I/II
NCT02660034	Extensive SCLC	BGB-290	BGB-A317 (PD-1)	I/II
NCT02944396	NSCLC	Veliparib	Nivolumab (PD-1) + Platinum therapy	I/II
NCT02734004	SCLC	Olaparib	Durvalumab (PD-L1) +/- bevacizumab	I/II
NCT03330405	NSCLC	Talazoparib	Avelumab (PD-1)	II
NCT03308942	NSCLC	Niraparib	Anti-PD-1	II
NCT03775486	NSCLC	Olaparib	Durvalumab (PD-L1)	II

*SCLC, small-cell lung cancer; NSCLC, non-small-cell lung cancer*.

PARP inhibitors are attractive not only in tumors with underlying homologous recombination deficiencies, but also in tumors associated with high levels of replication stress, such as SCLC. SCLC cell lines have shown upregulation of *BCL2* and elevated *PARP1* levels (66). Early preclinical data has found PARP inhibition sensitized SCLC to cisplatin therapy (67). The IOLite open label trial (NCT03307785) is testing the safety and tolerability of the combination of anti-PD-1 and niraparib with the proteasome inhibitor bevacizumab in chemotherapy-resistant advance solid tumors, including SCLC (68). This elevated expression of *PARP1* in SCLC is even being explored as a dynamic, non-invasive imaging modality to measure the drug-target engagement of PARP inhibitors (69).

## Gastrointestinal Cancers

The use of the combination immunotherapy and PARP inhibition is particularly interesting the gastrointestinal cancers. Notably, defective DNA mismatch repair and microsatellite instability, seen in Lynch Syndrome and 15% of colon cancer, have phenomenal responses of 25–80% to single-agent immune checkpoint blockade (4). Germline DDR mutations are seen in about 3% in gastric cancer (70), 3–17% in pancreatic cancer (71), and up to 8% in colorectal cancer (72) based on the gene involved and the population group. More relevant to the use of possible combinatorial PARP and checkpoint inhibition, acquired mutations in HRR are seen in about 28.9% in biliary, 20.9% in hepatocellular, 20.8% in gastroesophageal, 15.4% in pancreatic, and 15% in colorectal cancer patients (73). Like previously described cancers, a number of investigations are underway in the context of gastrointestinal tumors with intact and defective DNA repair and have reported responses in gastric and pancreatic cancers (74). The below clinical trials are evaluating the combination of PARP agents with PD1/PD-L1 inhibitors for a synergistic response with trials that are currently underway summarized in Table 6.

**Table 6:** Active combinatorial trials in gastrointestinal cancers.

**Trial ID**	**Cancer**	**PARP inhibitor**	**Immunotherapy**	**Phase**
NCT02484404	Colorectal	Olaparib	Atezolizumab (PD-L1) cediranib (VEGFR)	I/II
NCT02660034	•HER2-negative gastric •Gastroesophageal junction •Advanced pancreatic	BGB-290	BGB-A317 (PD-1)	I/II
NCT03851614	•Advanced pancreatic •dMMR colorectal	Olaparib	Durvalumab (PD-1)	I/II
NCT03404960	Advanced pancreatic	Niraparib	Nivolumab (PD-1) ipilimumab (CTLA-4)	I/II
NCT03637491	Advanced pancreatic	Talazoparib	Avelumab (PD-1)	I/II
NCT02734004	Relapsed gastric	Olaparib	Durvalumab (PD-L1) +/− bevacizumab	I/II
NCT03639935	Biliary	Rucaparib	Nivolumab (PD-1)	II

The open-label MEDIOLA phase II basket study evaluating olaparib in combination with durvalumab in patients with relapsing gastric cancer after platinum-based chemotherapy (75). A 4-weeks run-in with olaparib was done to collect biopsies followed by combination therapy with olaparib and durvalumab until disease progression. Among 40 patients included in the study, an ORR of 10% (2 in CR and 2 in PR) was reported with a DCR at 12 weeks of 26% (75). In the patient with controlled disease, the median duration of response about 11.1 months. Grade 3 and grade 4 adverse events were reported for 48 and 8% of treated patients, with 25% of patients developing immune-mediated adverse events. These events principally consisted of anemia (17.5%) and increased lipase (10%). The authors theorized the low 12-weeks disease control rate may have been a result of a high rate of disease progression during the 4-weeks run in treatment with olaparib, which excluded patients from the final evaluation.

The promise of immunotherapy has not become a reality for pancreatic cancers, with low response rates to checkpoint inhibitors (4). With the FDA approval of PARP inhibitors for maintenance therapy in patients with *BRCA*-mutated pancreatic cancer in late 2019, investigations are proceeding with broadening the therapy using combinatorial approaches (76). For instance, PARPVAX is a phase Ib/II study intended for locally advanced/metastatic pancreatic cancer patients who did not progress after a minimum of 16 weeks of platinum-based chemotherapy. Eligible patients will be in the niraparib with nivolumab (anti-PD-1) arm or niraparib with ipilimumab arm with the primary outcome measure of 6-months progression-free survival in both the arms. However, current trials are underway that move beyond comparing PARP and checkpoint inhibition by adding additional agents. A phase Ib/II study of doublet therapy avelumab with binimetinib (MEK inhibitor); or with the addition of talazoparib (PARP inhibitor) in locally advanced/metastatic RAS-mutant solid tumor patients (including metastatic pancreatic ductal adenocarcinoma) with disease progression after one line of therapy is currently under investigation (Table 6). Another combinatorial approach is seen in the DAPPER phase II basket combination study of durvalumab with olaparib or cediranib (VEGFR inhibitor) in advanced DNA mismatch-repair-proficient colorectal cancer, pancreatic adenocarcinoma, or leiomyosarcoma who failed standard therapy.

## Discussion

The combination of PARP inhibitor and immune checkpoint agents targeting PD/PD-L1, appears to be safe, potentially synergistic and may represent a compelling strategy in treatment for a variety of tumors. There is difficulty in the concluding of synergy between the use of PARP inhibitors and immunotherapy, as a number of clinical trials summarized are in (1) early phases safety trials, (2) utilize patients in advanced clinical stages or after failure of multiple therapies, and (3) compare response to standard chemotherapy regimens. Moreover, these combinatorial trials do not have multiple arms comparing the treatment with one agent and often differ in inclusion criteria from the single-agent PARP or immune checkpoint trials. In the context of HRR-deficient tumors, remarkable responses have been reported in ovarian (ORR 45–63%, DCR 73–81%) and breast cancer (ORR 53% DCR: 47%) (21, 34, 46). Underlying these observations is the assumption that PARP inhibitors may act to prime the tumor microenvironment and increase anti-tumor immune cells, acting like an adjuvant for immunotherapy. For tumors that harbor a known alteration in HRR genes, such as *BRCA1* or *BRCA2*, higher tumor mutational burden and a greater number of tumor-infiltrating lymphocytes has been seen in breast and ovarian cancer (77, 78). In these instances, the combination therapy seems to act by tipping the balance in favor of an anti-tumor immune response in the setting of a primed tumor microenvironment. This theory is supported by the observations of the CTLA-4 inhibition working in synergy with PARP inhibition in *BRCA1* deficient tumors (21).

As summarized in this article the open question remains on translating the efficacy of the combination of PARP inhibition with immune checkpoint blockade in cancers intact DNA repair machinery. The majority of the clinical trials currently underway are investigating that very question. Early reports have shown more modest ORR of 14–19% in breast (49), 10% in gastric (68), and 7% in prostate cancers (59). Despite the low ORR for prostate cancer, there was a 46% DCR for advanced, therapy-resistant, metastatic disease (59). Mechanistic investigations are hopeful, with findings that include PARP inhibition induces PD-L1 expression in homologous-proficient breast cancer (17, 19). PARP inhibition can also promote the accumulation of cytosolic DNA fragments due to unresolved DNA lesions, which in turn activates the DNA sensing cGMP-synthase-stimulator-of-interferon genes (cGAS-STING) pathway (79). This stimulates the production of type I interferons, and in turn, induces antitumor immunity that may be further enhanced by checkpoint inhibitor blockade, independent of BRCAness (80). Other strategies that act to further prime the tumor microenvironment before or concomitantly with immunotherapy are being evaluated, such as the use of radiation or chemotherapy (4).

Combining PARP inhibitors with immunotherapy has the potential to improve outcomes in variety of solid tumors but several challenges remain. Most notably, lack of predictable responses in most tumor types and delineation of patients that would benefit from combination vs. sequential therapies. Identifying biomarkers of DNA damage repair and immune responsiveness would have to be streamlined. Various factors such as tumor vs. liquid biopsy, somatic vs. germline mutations, PDL-1 positivity on tumor cells vs. immune cells, nature and degree of DNA damage can potentially impact on clinical outcomes. Additionally, challenges remain in identifying meaningful endpoints of such combinations therapy like overall survival vs. response rates. Duration and frequency of such combination therapy and associated impact on health care costs should be considered. Research efforts should be focused not only on evaluating safety and efficacy, but also on biomarkers that can accurately predict benefit from such combination. The preliminary results of various studies evaluating the efficacy of PARP inhibitors and immunotherapy are promising in various solid tumors. The final results of the ongoing phase III clinical trials will eventually determine their overall efficacy and clinical benefit for the patients.

## Author Contributions

PV conceptualized the review, PV and NB contributed equally. AC and RJ contributed to the significant portions of the manuscript. All authors were involved in writing and editing and approval of final manuscript.

## Conflict of Interest

The authors declare that the research was conducted in the absence of any commercial or financial relationships that could be construed as a potential conflict of interest.
